# Prevalence and factors associated with poor sleep quality among patients with hypertension in a primary care clinic: A cross-sectional study

**DOI:** 10.51866/oa.830

**Published:** 2025-06-13

**Authors:** Hong Wea Lau, Siew Mooi Ching, Abu Bakar Fatimah

**Affiliations:** 1 MD, MMed (Family Medicine), PhD, Department of Family Medicine, Faculty of Medicine and Health Sciences, Universiti Putra Malaysia, Serdang, Selangor, Malaysia.; 2 Malaysian Research Institute on Ageing, Faculty of Medicine and Health Sciences, Universiti Putra Malaysia, Serdang, Selangor, Malaysia.; 3 Department of Medical Sciences, School of Healthcare and Medical Sciences, Sunway University, Bandar Sunway, Malaysia.; 4 Research Centre of Excellence Nutrition and Non-communicable Diseases, Faculty of Medicine and Health Sciences, UPM, Malaysia. Email: sm_ching@upm.edu.my; 5 MBBS, MMed (Family Medicine), Klinik Kesihatan Kepala Batas, Butterworth, Pulau Pinang, Malaysia.; 6 MBBS, MMed (Family Medicine), Klinik Kesihatan Sungai Dua, Butterworth, Pulau Pinang, Malaysia.

**Keywords:** Sleep quality, Prevalence, Hypertension, Primary care, Malaysia

## Abstract

**Introduction:**

International research has consistently shown that individuals with hypertension experience significantly impaired sleep quality, but there is a lack of study in this field in Malaysia. This study aimed to assess the prevalence and associated factors of poor sleep quality among patients with hypertension in a primary care clinic in Malaysia.

**Methods:**

A cross-sectional study was conducted among patients with hypertension aged 18 years and above in a public health clinic from December 2023 to February 2024. Systematic random sampling was used to recruit participants. Sociodemographic data and relevant information were obtained using the Pittsburgh Sleep Quality Index; Depression, Anxiety and Stress Scale-21; and STOP-BANG questionnaire. The Statistical Package for the Social Sciences version 27 was used to conduct multiple logistic regression analysis of the determinants of poor sleep quality.

**Results:**

A total of 335 participants were recruited, yielding a 98.2% response rate. The mean participant age was 58 years, and 63% were women. The prevalence of poor sleep quality was 87.2%. Multiple logistic regression showed that anxiety symptoms (odds ratio [OR] = 12.279, 95% confidence interval [CI] = 1.573-95.883, P=0.017), low monthly household income (OR=3.573, 95% CI= 1.627-7.847, P=0.002), lack of exercise (0R=2.530, 95% CI=1.108–5.775, P=0.028) and risk of obstructive sleep apnoea (OR=2.521, 95% CI=1.176–5.402, P=0.017) were associated with poor sleep quality.

**Conclusion:**

Poor sleep quality is prevalent among patients with hypertension and is associated with adverse cardiovascular events. Increased awareness among primary care physicians would allow early identification of patients at risk for early intervention.

## Introduction

Hypertension is a prevalent chronic condition that significantly contributes to cardiovascular morbidity and mortality worldwide.^1^ In Malaysia, hypertension remains a major burden to the healthcare system, with 3 out of 10 Malaysians (6.4 million) being hypertensive according to the 2019 National Health Morbidity Survey.^2^ A meta-analysis has shown that patients with hypertension are more likely to have poor sleep quality than those without hypertension.^3^ The prevalence of poor sleep quality among patients with hypertension varies from 14.9% to 85.7% worldwide across different studies.^4-8^ In Asia, the prevalence of poor sleep quality among adult patients with hypertension ranges from 35.6%^6^ to 60.4%.^4^ Studies conducted in primary care or community settings have reported a prevalence of poor sleep quality among patients with hypertension of 55.7% in Brazil,^5^ 38.2% in Italy^8^ and 42.4% in Nigeria.^7^

Research has shown that sociodemographic characteristics and lifestyle factors play significant roles in sleep quality.^9^ Numerous studies have linked poor sleep quality with sociodemographic factors such as older age,^10^ female sex,^10^ lower educational level,^6^ lower income level^9^ and lifestyle habits including smoking^11,12^ and lack of exercise.^10,13^

Older adults, particularly those with hypertension, have been found to have a higher likelihood of having poor sleep quality, possibly due to age-related declines in circadian rhythm amplitude and reductions in melatonin secretion, both of which are essential for sleep regulation.^14^ In addition, female patients with hypertension are more prone to experiencing poor sleep than their male counterparts, likely due to the combined burden of household responsibilities and greater susceptibility to psychological distress.^15^ Unfavourable socioeconomic factors such as unemployment and low household income are associated with poor sleep quality, which can be attributed to financial stress and increased anxiety in patients belonging to lower income groups.^9^ Cigarette smoking has been strongly linked to sleep-wake cycle disruptions, particularly prolonged sleep onset latency due to the release of chemicals such as dopamine and serotonin, which promote wakefulness and interfere with REM sleep.^16^ In addition, other factors such as depression, increased anxiety or stress are also a significant contributor to poor sleep quality.^17^ Individuals with such psychological conditions usually experience sleep until the REM stage, without progressing to deep sleep state, due to decrease in serotonin levels or sleep-inducing chemicals.^10^ Conversely, regular physical activity has been identified as a protective factor against poor sleep quality. Exercise promotes better sleep through multiple mechanisms, including the release of endorphins, which reduce stress and anxiety, as well as improvement in circadian rhythm regulation and body temperature control, fostering greater relaxation and sleep efficiency.^18,19^

Poor sleep quality among patients with hypertension is associated with an increased risk of stroke and cardiovascular disease due to increased sympathetic nervous system activity, elevated blood pressure and impaired endothelial function.^20^ Furthermore, inadequate sleep has been shown to increase appetite, which leads to obesity and the development of diabetes.^21^ Therefore, understanding the factors contributing to poor sleep quality among patients with hypertension is important to improve the management of hypertension and achieve the target blood pressure control.

To date, limited studies have assessed the prevalence and associated factors of poor sleep quality among patients with hypertension in Malaysia. It is imperative to investigate poor sleep quality in patients with hypertension due to its significant impact on cardiovascular health. Hence, this study aimed to determine the prevalence of and factors associated with poor sleep quality among patients with hypertension in a primary care setting.

## Methods

### Study design

A cross-sectional study was conducted at Klinik Kesihatan Sungai Dua (KKSD) in Seberang Perai Utara (SPU), a district in Penang. This clinic covers four rural clinics and one community clinic. The population in SPU based on census 2010-2020 was 405,100.^22^

Patients aged 18 years who had registered to have hypertension at KKSD while on treatment follow-up for ≥6 months were included in this study. Pregnant patients, patients with selfreported disabilities preventing full participation in this study such as hearing/vision disabilities, patients with mental health problems or psychiatric disorders and patients unable to understand Malay or English were excluded from this study.

### Sample size calculation

The sample size was calculated using G*Power 3.1.9.7 based on the odds of having poor sleep quality among patients with hypertension and depression (2.03). The calculated sample size was 265 based on a power of 80%, a confidence interval (CI) of 95% and a P-value of 0.05. The final sample size was 332 after adjusting for a 20% non-response rate.

### Data collection and sampling method

Patients with hypertension who fulfilled the inclusion criteria from 1 December 2023 to 28 February 2024 were included in the study using the systematic random sampling technique. With a sampling interval of 2, the first patient was randomly selected using coin toss, with the numeric side designated as ‘2’ and the symbolic side as ‘1’. As the coin toss showed the numeric face, the first invited participant was patient number 2 in the list of patients with hypertension on follow-up for the day. Subsequently selected participants followed an arithmetic sequence, with a common difference of 2.

### Study tools

Our study used a set of interviewer-assisted questionnaire available in Malay and English, comprising four parts. The first part contained questions regarding sociodemographic and clinical characteristics of participants. The second part contained the Pittsburgh Sleep Quality Index (PSQI), whose Malay version was validated, with a Cronbach’s alpha coefficient of 0.74.^23^ The third part included the Depression, Anxiety and Stress Scale-21 (DASS-21), with its Malay version validated, with Cronbach’s alpha coefficients of 0.75, 0.74 and 0.79 for the depression, anxiety and stress subscales, respectively.^24^ The last part was the STOP-BANG questionnaire, which was filled in by the researcher only. The sensitivity, specificity and positive and negative predictive values of the Bahasa Malaysia version were 61.42%, 71.05%, 84.06% and 41.54%, respectively.^25^

The PSQI is a self-reported tool used to measure subjective sleep quality. It has a minimum score of 0 and a maximum score of 21. Global scores of <5 and ≥5 indicate good and poor sleep quality, respectively. In the previous validation study, this scoring accurately identified 88.5% of the patient group.^26^

In our study, the dependent variable was sleep quality, which was categorised as good (PSQI global score of <5) and poor (PSQI global score of ≥5). The independent variables were sociodemographic (i.e. age, sex, ethnicity, occupation, marital status, educational level and household income) and clinical characteristics (i.e. smoking status, alcohol drinking, exercise status, duration of hypertension, presence of comorbidities, body mass index, systolic blood pressure, diastolic blood pressure, risk of obstructive sleep apnoea [OSA] and psychological status including depression, anxiety or stress).

### Pilot study

A pilot study was conducted among 30 participants before the main data collection to identify potential issues with the questionnaire and recruitment process. The questionnaire was primarily completed by participants with the researcher’s assistance. No changes were made to the questionnaire after the pilot study. In this pilot study, the Cronbach’s alpha coefficient was 0.846 for the PSQI, 0.852 for the DASS-21 (0.777 for the depression domain, 0.498 for the anxiety domain and 0.762 for the stress domain) and 0.605 for the STOP-BANG questionnaire. The data gathered from the pilot study were excluded from our final study.

### Data analysis

Statistical analysis was conducted using the IBM Statistical Package for the Social Sciences version 26.0 (IBM Corp., Armonk, N.Y., USA). Descriptive analysis was initially performed to describe the characteristics of participants. We utilised the chi-square test for categorical data and an independent t-test or the Mann-Whitney U test for continuous data to ascertain associations between the dependent variable (sleep quality) and independent variables (sociodemographic and clinical characteristics). Simple logistic regression was conducted to identify potential determinants with a P-value of <0.25 for inclusion in subsequent analysis with multiple logistic regression. Finally, multiple logistic regression was performed, with the results presented as odds ratios (ORs) with 95%

CIs. A significance level of <0.05 was used to determine statistical significance.

## Results

A total of 341 eligible patients with hypertension attending KKSD were invited to participate in this study. Six patients withdrew after enrolment in this study. Ultimately, the study included 335 respondents, yielding a response rate of 98.2% (335 out of 341).

The majority of the respondents were aged 58.3 years (standard deviation= 10.25 years), were women (63.0%) and were of Malay ethnicity (76.1%). Most of them (80.6%) had a monthly household income of <RM 4850.0 - the specific cut-off value of monthly household income based on the Household Income and Basic Amenities Survey Report 2019 by the Department of Statistics Malaysia.^27^ Approximately 40.9% of the respondents did not perform any exercise. Additionally, more than half (56.7%) were obese, and almost one-third (31%) were overweight.

Among the respondents, 87.2% reported poor sleep quality. More than half (55.8%) were at risk of OSA. In terms of psychological status, 93.4%, 72.5% and 74.6% reported no depressive, anxiety and stress symptoms, respectively. Table 1 shows the participants’ sociodemographic characteristics, clinical characteristics and sleep quality.

**Table 1:** Descriptive analysis of the sociodemographic characteristics, clinical characteristics and sleep quality of the patients with hypertension (N=335).

**Item**		n	%	Mean	Standard deviation	Median	Interquartile range
**Sociodemographic characteristics**							
Age (year)				58.3	10.25		
Sex	Male	124	37.0				
	Female	211	63.0				
Ethnicity	Malay	255	76.1				
	Chinese	63	18.8				
	Indian	17	5.1				
Educational level	No formal education	36	10.7				
	Primary education	117	34.9				
	Secondary education	139	41.5				
	College/university education	43	12.8				
Marital status	Single	32	9.5				
	Married	278	83.0				
	Widowed	15	4.5				
	Divorced	10	3.0				
Occupation	Government sector	68	20.3				
	Private sector	101	30.1				
	Self-employed	34	10.1				
	Retiree	52	15.5				
	Unemployed	80	23.9				
Monthly household income	<RM 4850	270	80.6				
	RM 4850-10,959	61	18.2				
	>RM 10,959	4	1.2				
**Clinical characteristics**							
Systolic blood pressure (mmHg)						131.0	15.0
Diastolic blood pressure (mmHg)						74.0	12.0
Systolic blood pressure (mmHg)	<140	278	82.9				
	≥140	57	17.1				
Diastolic blood pressure (mmHg)	<90	309	92.2				
	≥90	26	7.8				
Duration of hypertension (year)						6.0	8.0
Comorbidity	Hypertension alone	131	39.1				
	With diabetes alone	40	11.9				
	With dyslipidaemia alone	102	30.4				
	With both diabetes and dyslipidaemia	48	14.3				
	With ischaemic heart disease alone	8	2.4				
	With chronic kidney disease alone	6	1.8				
Smoking status	Non-smoker and ex-smoker	271	80.9				
	Current smoker	64	19.1				
Alcohol drinking	Non-alcohol drinker	316	94.3				
	Alcohol drinker	19	5.7				
Exercise status	None	137	40.9				
	<150 minutes/week	179	53.4				
	≥150 minutes/week	19	5.7				
Body mass index (BMI)	Underweight (BMI of <18.5 kg/m^2^)	4	1.2				
	Normal (BMI of 18.5-22.9 kg/m^2^)	37	11.0				
	Overweight (BMI of 23-27.4 kg/m^2^)	104	31.0				
	Obese (BMI of ≥27.5 kg/m^2^)	190	56.7				
**Risk of OSA**							
**Not at risk of OSA (STOP-BANG score of <3)**		148	44.2				
**At risk of OSA (STOP-BANG score of >3)**		187	55.8				
**Psychological status**							
Depressive symptoms	Normal (score of 0–9)	313	93.4				
(DASS-21 depression score)	Mild (score of 10–13)	17	5.1				
	Moderate (score of 14–20)	3	0.9				
	Severe (score of 21–27)	2	0.6				
	Very severe (score of >27)	0	0				
Anxiety symptoms	Normal (score of 0–7)	243	72.5				
(DASS-21 anxiety score)	Mild (score of 8–9)	17	5.1				
	Moderate (score of 10–14)	41	12.2				
	Severe (score of 15–19)	16	4.8				
	Very severe (score of >19)	18	5.4				
Stress symptoms	Normal (score of 0–14)	250	74.6				
(DASS-21 stress score)	Mild (score of 15–18)	39	11.6				
	Moderate (score 19-25)	39	11.6				
	Severe (score of 26–33)	7	2.1				
	Very severe (score of >33)	0	0				
**Sleep quality**							
**Good sleep quality (PSQI score of <5)**		43	12.8				
**Poor sleep quality (PSQI score of ≥5)**		292	87.2				

The determinants of poor sleep quality were identified through initial analysis using simple logistic regression, followed by subsequent analysis using multiple logistic regression. The independent variables with a P-value of <0.25 were included in the multiple logistic regression analysis. A total of 10 variables were included: sex (P=0.001), educational level (P=0.094), monthly household income (P=0.007), duration of hypertension (P=0.078), smoking status (P=0.041), exercise status (P=0.006), BMI (P=0.038), risk of OSA (P<0.001), anxiety symptoms (P=0.004) and stress symptoms (P=0.006) (Table 2).

**Table 2:** Simple logistic regression of the associated factors of poor sleep quality among the patients with hypertension (N=335).

Variable		OR	**95% CI**		P-value
			Lower	Upper	
**Age**		1.015	0.983	1.047	0.367^↑^
Sex	Male	1			
	Female	0.545	0.264	1.124	0.001^*^
Ethnicity	Malay	1			
	Chinese	0.830	0.373	1.847	0.649^*^
	Indian	0.646	0.176	2.375	0.511^*^
Educational level	Tertiary education	1			
	Nontertiary education	2.009	0.887	4.548	0.094^*^
Marital status	Single	1			
	Married	0.902	0.3	2.716	0.855^*^
	Widowed^*^	-	0.000	-	0.999^*^
	Divorced	1.286	0.127	13.036	0.832^*^
Occupation	Employed	1			
	Unemployed and retiree	1.248	0.639	2.437	0.517^*^
Monthly household	<RM 4850 (B40)	2.593	1.291	5.206	0.007^*^
income	RM 4850-10,959 (M40) and >RM 10,959 (M40 and T20)	1			
SBP (mmHg)		0.990	0.963	1.018	0.478^^^
DBP (mmHg)		0.985	0.956	1.015	0.321^^^
Blood pressure control	Controlled hypertension	1			
	Uncontrolled hypertension	1.08	0.473	2.466	0.855^*^
**Duration of hypertension (year)**		1.054	0.994	1.118	0.078^^^
Comorbidity	Hypertension alone	1			
	Hypertension with other comorbidities	0.813	0.416	1.588	0.544^*^
Smoking status	Non-smoker and ex-smoker	1			
	Current smoker	3.521	1.053	11.768	0.041^*^
Alcohol drinking	Non-alcohol drinker	1			
	Alcohol drinker	1.267	0.282	5.688	0.757^*^
Exercise status	Exercise	1			
	No exercise	2.949	1.365	6.370	0.006^*^
BMI	Normal, underweight and overweight	1			
	Obese	1.991	1.040	3.810	0.038^*^
Risk of OSA	Not at risk of OSA	1			
	At risk of OSA	3.864	1.907	7.830	<0.001^*^
Depressive symptoms	No depression (normal)	1			
	At risk of depression^*^ (mild to very severe)	-	0.000		0.998^*^
Anxiety symptoms	No anxiety (normal)	1			
	At risk of anxiety (mild to very severe)	19.015	2.577	140.300	0.004^*^
Stress symptoms	No stress (normal)	1			
	At risk of stress (mild to very severe)	16.962	2.297	125.240	0.006^*^

* The OR could not be derived due to the presence of a zero value in a cell.

↑ Obtained via a t-test

* Obtained via the chi-square test

^ Obtained via the Mann–Whitney U test

The final model from multiple logistic regression revealed four factors associated with poor sleep quality among the patients with hypertension: anxiety symptoms (OR=12.279, 95% CI=1.573–95.883, P=0.017), low monthly household income (OR=3.573, 95% CI= 1.627–7.847, P=0.002), lack of exercise (OR=2.530, 95% CI=1.108–5.775, P=0.028) and risk of OSA (OR=2.521, 95% CI=1.176–5.402, P=0.017) (Table 3).

**Table 3:** Multiple logistic regression of the associated factors of poor sleep quality among the patients with hypertension (N=335).

Variable		aOR	95% CI		P-value
			Lower	Upper	
Anxiety symptoms	No anxiety (normal)	1			-
	At risk of anxiety (mild to very severe)	12.279	1.573	95.883	0.017
Monthly household income	<RM 4850 (B40)	3.573	1.627	7.847	0.002
	RM 4850–10,959 and >RM 10,959 (M40 and T20)	1			-
Exercise status	Exercise	1			-
	No exercise	2.530	1.108	5.775	0.028
Risk of OSA	Not at risk of OSA	1			-
	At risk of OSA	2.521	1.176	5.402	0.017
Smoking status	Non-smoker and ex-smoker	1			-
	Current smoker	1.796	0.412	7.830	0.435
BMI	Normal, underweight and overweight	1			-
	Obese	1.090	0.514	2.311	0.822
Educational level	Tertiary education	1			-
	Nontertiary education	1.916	0.708	5.182	0.200
**Duration of hypertension**		1.033	0.971	1.098	0.309
Sex	Male	1			-
	Female	1.518	0.523	4.403	0.443
Stress symptoms	No stress (normal)	1			-
	At risk of stress (mild to very severe)	5.922	0.746	47.019	0.092

* aOR: adjusted odds ratio

## Discussion

The prevalence of poor sleep quality among the patients with hypertension in our study was high at 87.2% (response rate: 98.2%). To the best of our knowledge, no prior study conducted worldwide has reported a higher prevalence of poor sleep quality among patients with hypertension. In our study, the mean patient age was 58.3±10.25 years. Similarly, a clear trend emerged across the literature indicating higher prevalence rates with increasing mean age. For instance, Birhanu et al. reported a prevalence of 35.5% at a mean age of 53.15± 11.54 years in Ethiopia,^13^ while Gou et al. found a prevalence of 60.4% at 63.89± 12.25 years in China.^28^ Our participants’ mean age closely matches that in the study conducted by Alebiosu et al. in Nigeria,^7^ who reported a prevalence of 42.4% at 58.15±9.65 years. Despite similar age profiles, our study recorded a substantially higher prevalence of poor sleep quality.

In the present study, the patients with mild-to-very severe anxiety symptoms were 12 times more likely to have poor sleep quality than those without anxiety symptoms (OR=12.279, 95% CI=1.573–95.883, P=0.017). This finding is in line with the report by Ayanaw et al., showing that patients with hypertension and anxiety were 1.89 times more likely to have poor sleep quality than those without anxiety.^10^ According to Manzar et al., both the presence of anxiety and increasing anxiety level among adults are linked to increased psychological stress, poor sleep hygiene and poor sleep quality.^29^ One possible explanation of poor sleep quality is that there is a decrease in serotonin levels or sleep-inducing chemicals during anxiety. Consequently, poor sleep quality might occur due to prolonged sleep onset latency, increased awakenings, more wakefulness during the night and fewer transitions into NREM sleep.^10^ Excessive anxiety levels in daily life may also disrupt neuroendocrine regulation, causing anxiety and tension, which ultimately affect sleep quality.^30^ Wide CIs of anxiety symptoms were found in this study, suggesting less precise estimates and greater uncertainty possibly due to the smaller sample size. Therefore, careful interpretation of this finding is warranted. Nevertheless, the high prevalence of poor sleep quality among our respondents underscores the importance of acknowledging this significant factor with its evident association.

The patients with low monthly household income were 3.5 times more likely to have poor sleep quality than those with middle and high household incomes in this study (OR=3.573, 95% CI= 1.627–7.847, P=0.002). This finding was significant, as 80.6% of our respondents fell into this income category. Most studies focusing on populations with hypertension have not established a significant relationship between income and sleep quality.^6,10,13^ For instance, Mariani et al. found no significant association between economic status and poor sleep quality in their study conducted in Indonesia.^6^ Individuals with low economic status could still achieve good sleep quality with adequate social support.^31^ However, the study by Anders et al. on the general population in Germany revealed that a higher gross household income was associated with better sleep quality.^32^ Additionally, Grandner et al. reported that more sleep complaints were associated with lower socioeconomic status in terms of income and employment.^33^ This might be attributed to the mental satisfaction associated with a higher quality of physical living environment. In Malaysia, Loke and Ching noted that patients with low monthly household income were four times more likely to have psychological distress than those with higher income, suggesting that low monthly household income is a significant life stressor, which could disrupt sleep.^34^

Our study revealed that the patients who never exercised were 2.5 times more likely to have poor sleep quality than those who exercised (OR=2.530, 95% CI=1.108-5.775, P=0.028). This is supported by the findings of Ayanaw et al. and Birhanu et al. showing a significant association between physical inactivity and poor sleep quality among patients with hypertension.^10,13^ A study conducted in Spain examined the impact of an aerobic exercise programme on physical activity, sedentary behaviour and sleep quality among individuals with hypertension. The results showed that exercise reduced sedentary behaviour while improving sleep quality among overweight or obese adults with hypertension.^35^ Regular exercise may contribute to improved sleep quality through various mechanisms, including reducing daytime sleepiness, enhancing sleep efficiency, increasing oxygen consumption and promoting weight loss.^36^

The patients at risk of OSA were also 2.5 times more likely to experience poor sleep quality than those not at risk (OR=2.521, 95% CI= 1. 176–5.402, P=0.017). In our study, the patients with a STOP-BANG score of >3 were considered to be at risk of OSA, coinciding with moderate and high risks of OSA.^37^ Similarly, Bacci et al. found that patients with hypertension at a high risk of OSA had poor sleep quality.^38^

### Strengths

To our best knowledge, this study is the first to investigate sleep quality in Malaysia among patients with hypertension and its associated factors. The study was conducted in a primary care clinic that covered a diverse study population with sociodemographic characteristics mirroring those of the multi-ethnic general population in Malaysia. In addition to exploring the sociodemographic and clinical features of patients with hypertension, our study examined the risk of OSA and psychological status using validated and useful research questionnaire tools, broadening the scope of the variable associations. The results of our study highlight the importance of detecting poor sleep quality among patients with hypertension.

### Limitations

Our study was limited to a primary healthcare clinic in a government setting. Thus, the findings cannot be generalised to other populations. Additionally, there may be bias in the self-reported items such as exercise, alcohol consumption and smoking status.

## Conclusion

Our study found a high prevalence of poor sleep quality among patients with hypertension. Given the negative impact of poor sleep quality on cardiovascular health, healthcare personnel may begin asking patients with hypertension regarding their sleep quality using validated tools to identify those at risk for further intervention.

## Data sharing statement

Our raw data are only available upon request.

**How does this paper make a difference in general practice?**

- Our results suggest that the prevalence of poor sleep quality is notably high among patients with hypertension.
- We recommend promoting appropriate mental health screening tests to screen patients with hypertension for psychological distress in primary care settings.
- It is also highly recommended to screen for sleep quality among individuals with hypertension, especially those with risk factors of poor sleep quality, and provide interventions if indicated, given the high prevalence of poor sleep quality in our study.
